# Capnographic monitoring using a novel mainstream system during endoscopic ultrasound and endoscopic retrograde cholangiopancreatography: A prospective randomized controlled trial

**DOI:** 10.1002/jhbp.12110

**Published:** 2025-01-21

**Authors:** Yoichi Takimoto, Eisuke Iwasaki, Masayasu Horibe, Seiichiro Fukuhara, Kazuhiro Minami, Shintaro Kawasaki, Tatsuhiro Masaoka, Haruhiko Ogata, Fateh Bazerbachi, Takanori Kanai

**Affiliations:** ^1^ Division of Gastroenterology and Hepatology, Department of Internal Medicine Keio University School of Medicine Tokyo Japan; ^2^ Center for Diagnostic and Therapeutic Endoscopy Keio University School of Medicine Tokyo Japan; ^3^ CentraCare, Interventional Endoscopy Program St. Cloud Hospital St. Cloud Minnesota USA

**Keywords:** capnography, conscious sedation, endoscopic retrograde cholangiopancreatography, endoscopic ultrasound, hypoxemia

## Abstract

**Background/Purpose:**

Insufficient studies exist on capnography efficacy during endoscopic ultrasound or endoscopic retrograde cholangiopancreatography, and no definitive conclusions have been drawn. To evaluate the feasibility and efficacy of a novel mainstream capnography using an over‐the‐biteblock end‐tidal CO_2_ (EtCO_2_) detector in decreasing the risk of hypoxemia during endoscopic ultrasound (EUS) and endoscopic retrograde cholangiopancreatography (ERCP).

**Methods:**

Patients undergoing EUS or ERCP with conscious sedation at a single Japanese center were randomized to a control or a novel capnography monitored (intervention) group in a 1:1 ratio. Hypoxemia correction maneuvers were pursued if the oxygen saturation decreased to <92% in the control or intervention group and if a 15‐s suspension of EtCO_2_ wave occurred in the intervention group. The primary outcome was the incidence of hypoxemic events, defined as oxygen saturation <90%, during the procedures. Secondary outcomes included technical feasibility of EUS and ERCP with the use of this novel over‐the‐biteblock monitor.

**Results:**

In total, 250 patients were enrolled without dropouts or missing data (control group: 125; capnography group: 125). There was no significant difference in the incidence of hypoxemia between the control and capnography groups (29.6% [37/125] vs. 26.4% [33/125]; *p* = .573). The estimated odds ratio was 0.925 (95% confidence interval: 0.708–1.208). The EtCO_2_ concentration was successfully captured without impeding endoscopic maneuvers from the beginning to the end of the procedure in all patients.

**Conclusions:**

Although the novel mainstream capnography with an over‐the‐biteblock EtCO_2_ detector captures the EtCO_2_ concentration in EUS or ERCP under conscious sedation, it does not lead to the prevention of hypoxemia.

## Graphical abstract

In this randomized controlled trial, Takimoto and colleagues demonstrated that the novel mainstream capnographic bite block detected the end‐tidal CO_2_ concentration in 250 participants without interfering with EUS and ERCP procedures. In these relatively healthy, non‐obese patients, capnographic monitoring did not reduce the incidence of hypoxemia during EUS and ERCP‐related procedures.

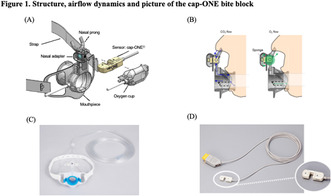

## 1 INTRODUCTION

**FIGURE 1:**
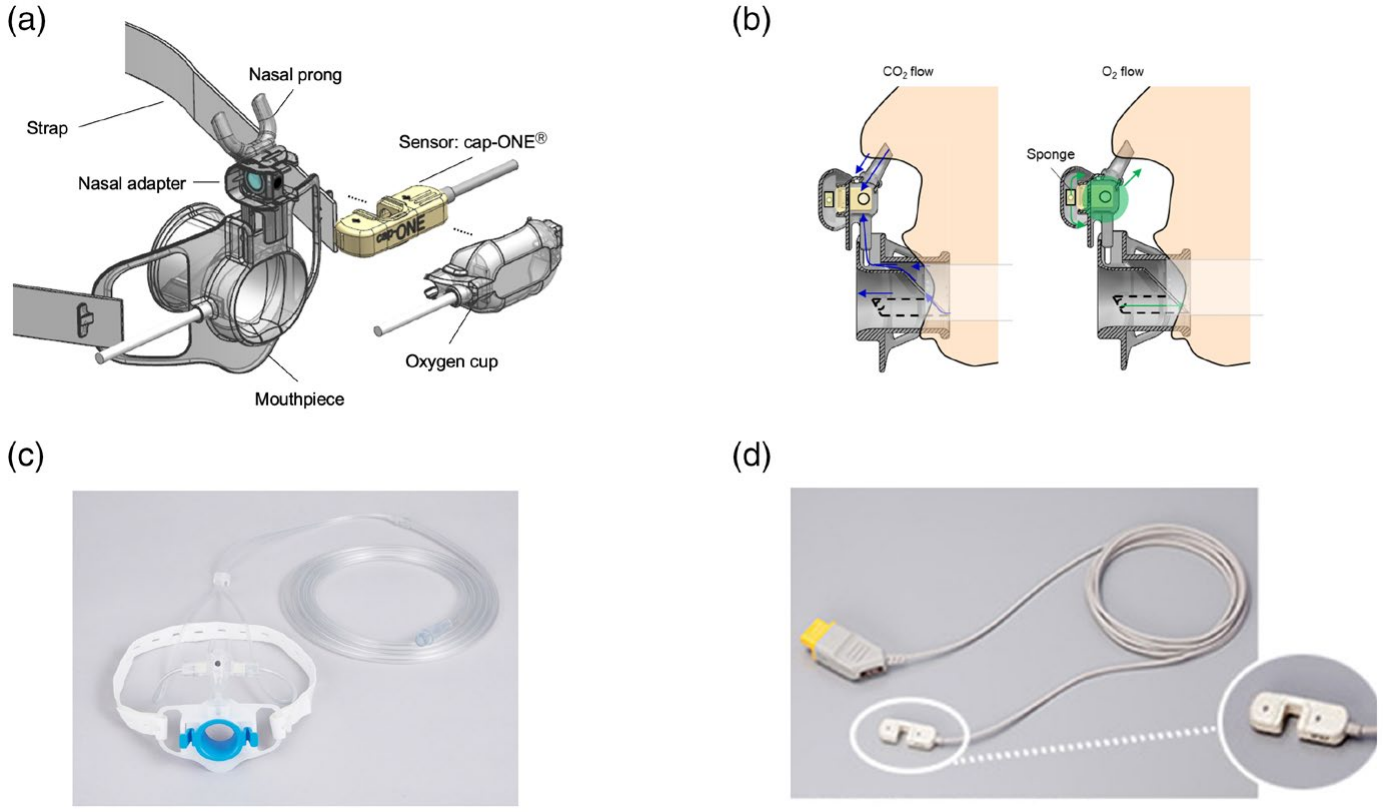
Structure and airflow dynamics of the cap‐ONE biteblock (TG‐980P; Nihon Kohden). (a) Structure of the cap‐ONE biteblock. The cap‐ONE biteblock system comprises a mainstream capnometer, nasal adapter, mouthpiece, and oxygen cup. (b) Flow of O_2_ and CO_2_ through the cap‐ONE biteblock. The cap‐ONE biteblock system is attached to the patient's face, as shown. (c) Image of biteblock and (d) sensor part.

**FIGURE 2:**
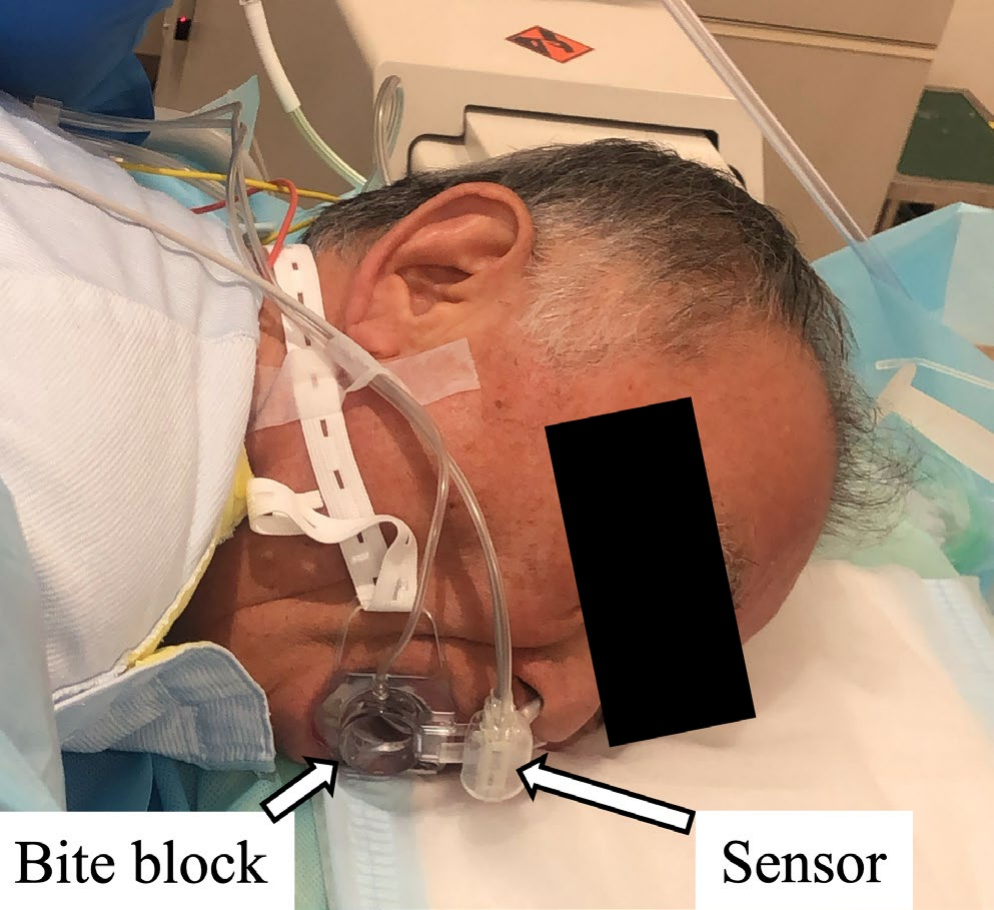
Cap‐One biteblock system with patient.

Compared with diagnostic upper and lower endoscopies, endoscopic retrograde cholangiopancreatography (ERCP) and endoscopic ultrasound (EUS) are considered complicated modalities requiring prolonged patient sedation time that necessitate safety measures to ensure patient stability during the procedure.^1^ While anesthesiology‐directed deep sedation is the norm for these procedures in the United States, endoscopist‐directed moderate conscious sedation (CS) is typically followed in Japan and other countries due to the considerations of cost and anesthesia staff availability. Hypoxemia is the most frequent adverse event associated with CS during endoscopic procedures.^2^ Previous studies have reported that hypoxemia occurred in 44%–69% of EUS or ERCP‐related procedures.^3^, ^4^ Although it has been reported that increasing oxygen flow itself prevents the development of hypoxemia,^5^ it seems to have a limited effect on reducing the incidence of hypoxemia following respiratory depression. Apnea caused by respiratory depression or comorbid respiratory dysfunction, such as chronic obstructive pulmonary disease, is thought to give rise to hypoxemia. Therefore, early detection of apnea may reduce hypoxemia incidence, thereby increasing the safety of CS during EUS or ERCP.^6^ Conventional percutaneous CO_2_ monitors are limited by their poor reliability during endoscopy.^7^, ^8^ End‐tidal capnography is a non‐invasive method of detecting depressed respiratory activity via CO_2_ measurements throughout the respiratory cycle. It has previously been demonstrated that end‐tidal CO_2_ (EtCO_2_) measurement during endoscopic procedures using sidestream type capnography was difficult due to blockage of the sampling tube with saliva and inability to detect shallow breathing or tachypnea.^3^, ^4^ To address this issue, we recently developed a novel, mainstream capnographic over‐the‐biteblock system (Cap‐One biteblock system, Nihon Kohden, Tokyo, Japan)^9^, ^10^ to detect nasal and oral tidal flow without the need for a sampling tube, thereby avoiding the limitations of sidestream capnography. The development of a mainstream style capnometer system that can be used with endoscopes has been considered difficult due to the large size of the sensor part, but we have succeeded in miniaturizing it. The cap‐ONE bite block system can deliver oxygen while measuring mainstream EtCO_2_ during endoscopic procedures. It comprises the mainstream capnometer, nasal adapter, oxygen cup, and mouthpiece. The nasal adapter collects exhaled nasal flow into a measurement cell, while the oxygen cup delivers oxygen through the patient's nose and has a sponge to scatter oxygen for accurate CO2 monitoring. The mouthpiece has a double‐layer conduit to open the patient's mouth as well as to collect oral exhaled CO2. It also has an oxygen port, which can supply oxygen through the mouth. The novel biteblock is made of polyester resin and measures 37.5 mm wide and 36.5 mm high, with a minimum opening diameter of 20.8 mm and a depth of 33.5 mm. It is about 3 mm larger in both width and height than the normally used bite block. The price of reusable sensor part is 440000 Japanese Yen and the disposable biteblock part is 2400 Japanese Yen each (Figures 1 and 2).

We have previously reported a pilot study demonstrating the feasibility of this system to reliably detect EtCO_2_ in all participants.^11^ This method enables the characterization and monitoring of respiratory activity, such as apnea episodes, respiratory rate, EtCO_2_, waveform shape, and breathing rhythm.

There have been two previous randomized controlled trials (RCT) with conflicting results regarding the efficacy of capnography during EUS or ERCP,^3^, ^4^ with no conclusions reached on its effectiveness. Furthermore, there are insufficient studies to validate the effectiveness of mainstream capnography during endoscopy. In the present RCT, we aimed to evaluate the efficacy of this novel biteblock capnography in detecting apnea episodes to prevent hypoxemia during EUS and ERCP‐related procedures.

## 2 METHODS

### 2.1 Study design and setting

Patients in Keio University Hospital undergoing ERCP or EUS‐related procedures with CO_2_ insufflation under moderate CS were enrolled. The exclusion criteria included (1) age <18 years, (2) emergency procedures, (3) baseline use of oxygen or non‐invasive ventilation devices, (4) oxygen saturation (SpO_2_) <90% under room air, (5) American Society of Anesthesiologists physical status (ASA‐PS) class of IV or V, (6) the biteblock could not be installed, and (7) inability to give informed consent. A duodenoscope (JF‐260V or TJF‐260V; Olympus Corp. Tokyo, Japan) was used to perform ERCP, and EUS was performed using an echoendoscope (UCT‐260 or UE‐260; Olympus Corp. Tokyo, Japan). ERCP was performed in the prone position and EUS was performed in the left lateral position. Flunitrazepam 0.2 mg or midazolam 2.5 mg and pethidine 35 mg were used; however, older patients (>80 years) or those with severe, pre‐existing comorbidity received a lower initial dose. The dosage was adjusted according to the depth of sedation based on the Ramsay sedation scale score of 4–5.^12^ Once the targeted sedation level was achieved, it was maintained by repeated administration of flunitrazepam or midazolam. EtCO_2_, SPO_2_, heart rate, and spontaneous blood pressure were monitored continuously during the procedures using the newly developed mainstream capnograph in the study group. The accuracy of the total respiratory count using waveform analysis was evaluated using dedicated software (Nihon Kohden). Patients were randomly assigned in a 1:1 ratio to either the control or capnography group before the procedure. A randomization table was constructed by an independent statistician, and the assignments were sealed in sequentially numbered, identical, opaque envelopes. Only patients and a researcher analyzing continuous recorded EtCO_2_ data were blinded to the randomization schedule, which was provided by a staff member who was uninvolved in the procedures. However, the endoscopists were not blinded. The study was conducted in compliance with the CONSORT guidelines for reporting RCTs, was approved by the ethics committee of Keio University Hospital (approval no. 20170074), and registered with the University Hospital Medical Information Network (registration no. UMIN 000029407).

### 2.2 Interventions

All patients received the Cap‐One biteblock system (Figure 1) during the endoscopic procedures to ensure a blinded group assignment and to calculate the success rate of measurements using the new system. However, devices in the control group were set to hide the EtCO_2_ waves and silent apnea alarm. Moreover, in this group, the endoscopic team was prepared to provide hypoxemia correction maneuvers only when the SpO_2_ monitor alarm indicated a level <92%. In the intervention group, the correction maneuvers were in response to a 15‐s suspension of EtCO_2_ wave (absence of respiratory activity denoted by a flat line on the capnometer for 15 s), as described in previous studies,^3^, ^4^ as well as a decrease in SpO_2_ to <92%. Hypoxemia correction maneuvers consisted of (i) rousing the patient; (ii) starting oxygen supplementation (2 L/min) and continued until awakening from sedation; (iii) increasing oxygen supplementation to 4 L/min; (iv) withholding medication; and (v) stopping the procedure. After interventions (i) and (ii) were implemented, (iii) and (iv) were performed until a SpO_2_ of >90% or EtCO_2_ elevation was achieved. A medical doctor was designated as the research assistant for each procedure, and serial monitoring (time, type of alarm, action taken, etc.) was recorded on the case report form (CRF). If hypoxic or apnea alarms occurred, research assistants checked for malfunctioning of the bite block or SpO_2_ detector and responded according to the protocol described above. Artifacts and temporary measurement failures were listed on the CRF with the time and content. We considered the CRF monitoring results during data analysis.

### 2.3 Patient characteristics

Patient characteristics (age, sex, height, weight, body mass index [BMI], smoking history, alcohol consumption, regular use of sedatives, medical history, ASA‐PS classification, and baseline SpO_2_) and procedural details (procedure type, indication, procedural time, total flunitrazepam, midazolam and pethidine hydrochloride dosage, and physician's skill level) were recorded in a spreadsheet by the endoscopist at the end of the procedure.

### 2.4 Outcome measures and definition

The primary outcome was the incidence of hypoxemia, defined as a decrease in SpO_2_ to <90% as measured using a finger pulse oximeter. The secondary outcomes were apnea, defined as a 15‐s suspension of EtCO_2_ elevation on capnography; oxygen saturation <92%; bradycardia (heart rate < 50/min); hypotension (systolic blood pressure < 90 mmHg); adverse events related to the bite block system; technical success of EUS/ERCP procedural steps; patient satisfaction with endoscopy experience (numerous analog scale [NAS] 0–10); patient cooperation as rated by the endoscopist (NAS 0–10); assisted ventilation, such as continuous or bilevel positive airway pressure; death; and procedural time.

### 2.5 Statistical analysis

The study statistician calculated the sample size prior to recruitment. Hypoxemic events were assumed to occur in 40% of the control group based on previous articles,^3^, ^4^; thus, reduction to 20% by capnographic monitoring was considered clinically relevant. The sample size necessary to demonstrate the expected difference with a two‐sided significance level of 5% and statistical power of 90% was 125 patients per group (250 patients in total) after allowing for a 5% dropout rate. Primary and secondary analyses were performed for all randomized patients. The primary outcome was compared using the two‐sample *t*‐test. For the secondary outcomes, continuous variables were compared using the two‐sample *t*‐test, and categorical outcomes were analyzed using the *χ* ^2^ or Fisher's exact test, depending on the expected value. All statistical analyses were performed using SPSS version 25 (IBM Corp., Armonk, New York, USA).

## 3 RESULTS

### 3.1 Study population and feasibility of capnography

**FIGURE 3:**
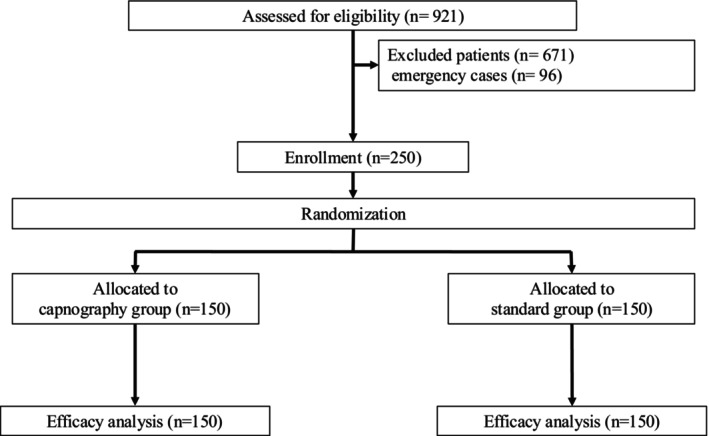
Flow chart of trial enrolment, randomization, and analysis.

In total, 346 patients who underwent EUS and ERCP‐related procedures were screened, and 250 patients (58.4% male) were enrolled (control group: *n* = 125; capnography group: *n* = 125) between February 2018 and February 2019. The trial ended after the specified number of participants was registered. Figure 2 presents the study flow. Table 1 summarizes the patients' baseline characteristics. Although all patients were randomized, there was a significant difference in age between the groups (capnography: 65.4 vs. control: 61.9; *p* = .032). There were no dropouts or missed recorded data in either group. No measurement failure was detected, and the EtCO_2_ concentration was successfully captured from the beginning to the end of the procedure in all patients. There was no damage to the biteblock observed (Figure 3, Table 1).

**TABLE 1:** Patient characteristics.

	Total (*n* = 250)	Capnography group (*n* = 125)	Control group (*n* = 125)	*p*‐value
Age, years, mean (SD)	63.6 (13.2)	65.4 (12.8)	61.9 (13.5)	.032
Sex (male), no. (%)	146 (58.4)	75 (60)	71 (56.8)	.608
Height, cm, mean (SD)	163 (9.07)	163 (8.87)	163 (9.31)	.929
Weight, kg, mean (SD)	60.0 (11)	60.3 (12.4)	59.7 (11.6)	.769
Body mass index, kg/m^2^, mean (SD)	22.2 (3.22)	22.3 (3.18)	22.2 (3.28)	.869
Smoking				.646
Never, no. (%)	120 (48)	63 (50.4)	57 (45.6)	
Current, no. (%)	30 (12)	13 (10.4)	17 (13.6)	
Ex‐smoker, no. (%)	100 (40)	49 (39.2)	51 (40.8)	
Alcohol consumption				
Daily consumption, no. (%)	52 (20.8)	28 (22.4)	24 (19.2)	.533
Regular consumption, no. (%)	168 (67.2)	87 (69.6)	81 (64.8)	.419
Current and previous drug abuse, no.(%)	0 (0)	0 (0)	0 (0)	
Regular narcotic/sedative use, no.(%)	31 (12.4)	15 (12)	16 (12.8)	.848
Heart disease, no. (%)	45 (18)	24 (19.2)	21 (16.8)	.621
Lung disease, no. (%)	17 (6.8)	6 (4.8)	11 (8.8)	.142
Renal disease, no. (%)	19 (7.6)	10 (8)	9 (7.2)	.811
Liver disease, no. (%)	23 (9.2)	14 (11.2)	9 (7.2)	.274
Neurologic/psychiatric disease, no. (%)	14 (5.6)	10 (8)	11 (8.8)	.820
Sleep apnea, no. (%)	4 (1.6)	3 (2.4)	1 (0.8)	.313
ASA‐PS class				.406
1, no. (%)	165 (66)	81 (64.8)	84 (67.2)	
2, no. (%)	78 (31.2)	42 (33.6)	36 (28.8)	
3, no. (%)	7 (2.8)	2 (1.6)	5 (4)	
Type of procedure				.959
ERCP, no. (%)	68 (27.2)	33 (26.4)	35 (28)	
EUS, no. (%)	164 (65.6)	83 (66.4)	81 (64.8)	
EUS FNA, no. (%)	18 (7.2)	9 (7.2)	9 (7.2)	
Indications				.981
CBD stone, no. (%)	21 (8.4)	9 (7.2)	12 (9.6)	
Malignant biliary stenosis, no. (%)	27 (10.8)	13 (10.4)	13 (10.4)	
Pancreatic carcinoma, no. (%)	25 (10)	12 (9.6)	13 (10.4)	
IPMN, no. (%)	74 (29.6)	38 (30.4)	37 (29.6)	
Chronic pancreatitis, no. (%)	12 (4.8)	6 (4.8)	7 (5.6)	
Others, no. (%)	91 (36.4)	47 (37.6)	43 (34.4)	
Baseline oxygen saturation, mean, SD	97.5 (1.6)	97.6 (1.4)	97.4 (1.7)	.657
Physician				.183
Trainee <3 years, no. (%)	23 (9.2)	9 (7.2)	14 (11.2)	
3–10 years, no. (%)	204 (85.6)	108 (86.4)	96 (76.8)	
Master >10 years, no. (%)	23 (9.2)	8 (6.4)	15 (12)	
Midazolam, no. (%)	132 (52.8)	59 (47.2)	73 (58.4)	.076
Total dose of midazolam (mg)		3.9 (1.9)	4.31 (1.9)	.132
Flunitrazepam, no. (%)	104 (41.6)	54 (43)	50 (40)	.608
Total dose of flunitrazepam (mg)		0.39 (0.22)	0.462 (0.40)	.671
Pethidine hydrochloride, no. (%)	224 (89.6)	113 (90.4)	111 (88.8)	.679
Total dose of pethidine hydrochloride (mg)		32 (11)	30.5 (12)	.308
Sedation addition, no. (%)		66 (52)	78 (62)	.125

Abbreviations: ASA‐PS, American Society of Anesthesiologists physical status; CBD, common bile duct; ERCP, endoscopic retrograde cholangiopancreatography; EUS, endoscopic ultrasonography; FNA, fine needle aspiration; IPMN, intraductal papillary mucinous neoplasm; SD, standard deviation.

### 3.2 Outcomes

There was no significant difference in the primary outcome of hypoxemia incidence between the control (*n* = 37, 29.6%) and capnography (*n* = 33, 26.4%) groups (*p* = .573). The estimated odds ratio was 0.925 (95% confidence interval [CI]: 0.708–1.208). Table 2 shows the secondary outcomes. The novel device did not hinder endoscopic maneuverability during EUS/ERCP, and there were no technical failures of EUS/ERCP procedures. There was no significant reduction in any of the secondary outcomes in the capnography group. There was no significant difference in patient satisfaction with the endoscopy, patient cooperation as rated by the endoscopist, number or frequency of apnea episodes, SpO_2_ <92%, bradycardia, hypotension, procedural parameters, or adverse events between the two groups. Patient satisfaction (6.0 vs. 6.2; *p* = .575) and patient cooperation (6.6 vs. 6.7; *p* = .534) rated by the endoscopist with sedation did not differ between the two groups (Table 2).

**TABLE 2:** Comparison of outcomes.

	Total (*n* = 250)	Capnography group (*n* = 125)	Control group (*n* = 125)	*p*‐value
Primary outcome				
Hypoxemia, *n* (%)	70 (28)	33 (26.4)	37 (29.6)	.573
Secondary outcomes				
Minimum SpO_2_, mean (SD)	90.6 (3.6)	90.6 (3.9)	90.6 (3.3)	.638
Oxygen saturation ≤92%, *n* (%)	147 (58.8)	71 (56.8)	76 (60.8)	.521
O_2_ supplementation, *n* (%)	150 (60)	77 (61.6)	73 (58.4)	.606
Apnea, *n* (%)	130 (52)	63 (50.4)	67 (53.6)	.613
Procedural time, mean (SD)	32.2 (13.9)	32.5 (15.9)	31.9 (11.8)	.719
Patient cooperation rated by an endoscopist	6.6 ± 2.1	6.7 ± 2.2	6.6 ± 2.1	.534
Patient satisfaction	6.1 ± 2.8	6.2 ± 2.8	6.0 ± 2.8	.575
Technical success of EUS/ERCP, *n* (%)	250	125	125	n.a.
Bradycardia, *n* (%)	19 (7.6)	10 (8)	9 (7.2)	.811
Hypotension, *n* (%)	16 (6.4)	9 (7.2)	7 (5.6)	.605
Adverse events	0 (0)	0 (0)	0 (0)	
Ventilation	0 (0)	0 (0)	0 (0)	
Mortality	0 (0)	0 (0)	0 (0)	

*Note*: 10 = excellent; 0 = poor.

Abbreviations: ERCP, endoscopic retrograde cholangiopancreatography; EUS, endoscopic ultrasonography; SD, standard deviation SpO_2_, oxygen saturation.

### 3.3 Adverse event

There were no serious adverse events, such as death or respiratory failure, in either group.

## 4 DISCUSSION

To the best of our knowledge, this is the first RCT to examine the use of a novel mainstream capnography device during EUS or ERCP procedures in patients undergoing moderate CS. The capnography system achieved complete monitoring of all data during the procedures for the entire cohort. Although the present study did not demonstrate statistically significant benefits in preventing hypoxemia using this capnography system during ERCP or EUS with CS despite the large sample size (beta power = 0.9), the study is important as it introduces the feasibility of this system in all randomized patients without preventing adequate endoscopic examination. Although our findings suggested that routine capnography monitoring does not add considerable benefit to low‐risk patients with low BMI (97% of enrolled patients were categorized as having ASA‐PS class I and II; no patients were obese; and 84% had BMI <25 kg/m^2^), the capnography system did not hinder the maneuverability of the relatively large duodenoscope and stiffer echoendoscope.

End‐tidal capnography is reportedly useful in the intensive care unit,^13^ but its utility during routine endoscopic procedures has not been confirmed,^14^, ^15^, ^16^ The European Society of Gastrointestinal Endoscopy guidelines recommend capnographic monitoring during the administration of propofol by a non‐anesthesiologist in specific situations, including the treatment of high‐risk patients, intended goal of deep sedation, and lengthy procedures. In these guidelines, the routine capnography procedure for detecting EtCO_2_ during endoscopy with sedation was not recommended because of the lack of any demonstrable benefit. Capnography was recommended for deep sedation based on low‐quality evidence from the American Society for Gastrointestinal Endoscopy (ASGE)^1^ and the Australian Gynecological Endoscopy and Surgery Society.^17^ The use of capnography with EUS and ERCP‐related procedures is not recognized as a standard monitoring method in the ASGE guidelines due to the low quality of the evidence. Indeed, the results of our large, randomized, controlled study support the existing recommendations of these guidelines.^1^, ^17^, ^18^ Monitored anesthesia care (MAC) includes all aspects of anesthesia care – a preprocedure assessment and optimization, intraprocedure care and postprocedure management that is inherently provided by a qualified anesthesia provider as part of the bundled specific service. The American Society of Anesthesiologists has defined MAC as a specific anesthesia service performed by a qualified (trained) anesthesia provider, for a diagnostic or therapeutic procedure. The introduction of MAC to ERCP and EUS‐related procedures would be considered in the future in terms of improving the safety the procedures, which are expected to become more complicated and difficult. However, the added benefit of capnography in patients at a higher risk, who typically undergo MAC, is yet to be evaluated. Indeed, the depth levels of MAC procedures may be significant, at times becoming general anesthesia without airway protection.^19^ Our current study lays the foundation for future studies, given the versatility of this new device during complex EUS/ERCP procedures. When designing a RCT in patients at a higher risk, it is important to know a priori that the use/application of this device will not hinder the critical steps of the proposed endoscopic intervention in more tenuous patients, which this RCT successfully demonstrates.

A previous trial comparing capnographic and oximetric monitoring in patients undergoing EUS or ERCP revealed that the frequency of hypoxemia was significantly lower in patients with capnography (46.0% vs. 69.1%; *p* < .001).^3^ However, another trial produced contradictory results (38.0% vs. 44.4%; *p* = .314)^4^; their per‐protocol analysis, excluding cases of mismeasurement caused by technical problems with capnography, showed a significantly lower frequency of hypoxemia in the capnography group (31.5% vs. 44.4%; *p* = .048).^16^ These conflicting results are thought to arise from the relative inaccuracy of sidestream capnography. A per‐protocol analysis in the latter study, which excluded measurements deemed inaccurate, underscored the efficacy of capnography.

To test this hypothesis, we developed a novel mainstream capnographic biteblock system (Cap‐One biteblock system, Nihon Kohden).^7^, ^9^ Mainstream capnography is considered superior to sidestream capnography due to several advantages.^19^, ^20^, ^21^ The disadvantages of the current sidestream system include delayed response due to the length of the sampling tube, obstruction of the tube by water or saliva, and measurement inaccuracies caused by the high oxygen flow. The rate of disruption of capnography in two previous RCTs was reportedly 13.3% (35/263)^3^ and 9.1% (11/121).^16^ On the other hand, mainstream capnography can measure exhaled CO_2_ directly around the mouth and nose without a delayed response and can prevent obstruction of the tube by water or saliva. Therefore, mainstream capnography is more reliable and accurate. Importantly, this system did not hinder adequate endoscopic examinations and therapies.

In the present study, EtCO_2_ was successfully measured in all patients during the procedure under CO_2_ insufflation. However, interventions targeted following a 15‐s episode of apnea did not improve hypoxemia, although the CO_2_ level was measured. Capnography may have shown no benefits for hypoxemia, possibly due to the relatively healthy status of patients and the lack of overweight or obese individuals. In addition, the sedative dose used was determined according to the patients' general condition and age by endoscopists rather than anesthesiologists. Moreover, due to the regulations at the study center, propofol was not used. Sedation was mild, which was reflected in the mean scores of 6.1 ± 2.8 and 6.6 ± 2.1 on an 11‐point scale for patient satisfaction and patient cooperation rated by the endoscopist, respectively, which were lower than previously reported scores (9.2 and 8.0).^4^ The relatively shallow depth of sedation achieved with non‐propofol sedatives administered by non‐anesthesiologists compared to previous studies might have reduced the frequency of respiratory depression detectable by capnography.

The analysis of all waveform data from the 125 patients in the control group showed that the susceptibility of apnea alone to subsequent hypoxemia was 49%, indicating that most patients with hypoxemia did not receive any intervention and that it was difficult to prevent hypoxemia by monitoring apnea alone. Respiratory abnormalities preceding hypoxemia may include changes in respiratory status, such as the respiratory rate and CO_2_ concentration, as well as apnea. Thus, interventions triggered by signs other than respiratory arrest for 15 seconds may reduce the rate of secondary hypoxemia. A more accurate definition of respiratory abnormalities preceding hypoxemia can be formed by analyzing patients' respiration data.

In summary, the greatest advantage of the novel capnometer system developed in this study is the ability to monitor respiration during all procedures without interfering with the procedure. Previous studies have reported that capnography reduces hypoxemia during EUS/ERCP except in cases of poor measurements. Combined with the results of this study, capnography may make EUS and ERCP procedures safer in patients in poor general condition, obese patients, and patients with deep sedation. In other words, the use of the mainstream capnometer may be recommended in patient groups at high risk for respiratory depression due to sedation, since accurate monitoring of respiratory status may prevent hypoxemia.

This study had some limitations. First, it was performed at a single institution and was single‐blinded, possibly introducing an observer bias. Moreover, generally, the envelope method adopted in this RCT is not sufficiently high quality. A multicenter, double‐blinded study is required to determine the generalizability of the findings. However, the selected patients were randomized, and there is no other bias in both groups. Second, the incidence of hypoxemia was estimated as high as 40% based on previous Western studies, the actual incidence was 28%. Possible reasons for this difference include the relative better health of the patient population and the type of sedative administered.

In conclusion, the newly designed mainstream capnographic bite block detected the EtCO_2_ concentration in 250 participants without interfering with adequate endoscopic examinations and interventions in EUS and ERCP procedures. In relatively healthy, non‐obese patients, capnographic monitoring does not reduce the incidence of hypoxemia during EUS and ERCP‐related procedures, and future studies should evaluate the role of this technology in patients at a higher risk who are undergoing deep sedation with MAC.

## AUTHOR CONTRIBUTIONS

YT and EI made substantial contributions to the conception of the work. YT, MH, and EI made significant contributions to the data analysis and interpretation. YT, MH, and EI made significant contributions to the design of the work and the interpretation of data. YT drafted the original manuscript. MH, EI, FB, and other authors substantially contributed to the revision of the manuscript drafts. All authors approved the submitted version of the manuscript and agree to be accountable for any part of the study.

## CONFLICT OF INTEREST STATEMENT

This study was conducted conjointly with Nihon Kohden Co., Ltd., who contributed to the development of the bite blocks and monitors used. The authors declare no conflict of interest for this article.
